# Textile Electrodes: Influence of Knitting Construction and Pressure on the Contact Impedance

**DOI:** 10.3390/s21051578

**Published:** 2021-02-24

**Authors:** Luisa Euler, Li Guo, Nils-Krister Persson

**Affiliations:** 1 Polymeric E-Textiles, Department of Textile Technology, University of Borås, SE-501 90 Borås, Sweden; luisa.euler@hb.se (L.E.); li.guo@hb.se (L.G.); 2 Smart Textiles Technology Lab, Smart Textiles, University of Borås, SE-501 90 Borås, Sweden

**Keywords:** impedance spectroscopy, textile electrodes, electrode construction, pressure influence, electrical stimulation, biomedical signal monitoring

## Abstract

Textile electrodes, also called textrodes, for biosignal monitoring as well as electrostimulation are central for the emerging research field of smart textiles. However, so far, only the general suitability of textrodes for those areas was investigated, while the influencing parameters on the contact impedance related to the electrode construction and external factors remain rather unknown. Therefore, in this work, six different knitted electrodes, applied both wet and dry, were compared regarding the influence of specific knitting construction parameters on the three-electrode contact impedance measured on a human forearm. Additionally, the influence of applying pressure was investigated in a two-electrode setup using a water-based agar dummy. Further, simulation of an equivalent circuit was used for quantitative evaluation. Indications were found that the preferred electrode construction to achieve the lowest contact impedance includes a square shaped electrode, knitted with a high yarn density and, in the case of dry electrodes, an uneven surface topography consisting of loops, while in wet condition a smooth surface is favorable. Wet electrodes are showing a greatly reduced contact impedance and are therefore to be preferred over dry ones; however, opportunities are seen for improving the electrode performance of dry electrodes by applying pressure to the system, thereby avoiding disadvantages of wet electrodes with fluid administration, drying-out of the electrolyte, and discomfort arising from a “wet feeling”.

## 1. Introduction

Smart textiles are textiles offering a plurality of functions that can be achieved by employing or integrating other technologies, foremost of these electronics [1,2]. By this, smart textiles find use in various application fields, including protection and security, energy, and transportation, and not least the healthcare sector. A wide variety of sensors and actuators based on textiles are possible today, most of which are based on incorporation of electrical conductivity, which can be realized by various means, e.g., by conductive particles in inks or coating pastes, or by conductive fibers or yarns to construct fabrics and nonwovens [3,4]. Based on this technology, current research is investigating possibilities for manufacturing textile surface electrodes [5,6,7,8,9,10], also referred to as textrodes, to be used for instance in the healthcare sector, providing opportunities for home-based electrotherapy and self-administered monitoring of body functions [11,12,13,14,15]. In signal monitoring, electrodes are utilized for measuring body functions such as heart and muscle activity or breathing rate, as well as bioimpedance or body temperature [16,17]. Within this, the electrode functions to obtain a signal by recording the body surface potential and transforming it into an electrical current, which means the performance focus is on retrieving a good signal quality [18]. In electrotherapy on the other hand, electrodes are employed to inject current into the human body, thereby activating excitable tissue, i.e., muscles and/or nerves, to improve muscle strength, achieve cortical re-mapping, or suppress pain sensations [19,20,21,22]. Thus, in this field, the focus is on injecting a defined current into the body. Even though stimulation and monitoring electrodes have some differing requirements due to their end use, similar constructions can be used in both cases, having the requirement of a low impedance and low impedance variation [23].

**Figure 1:**
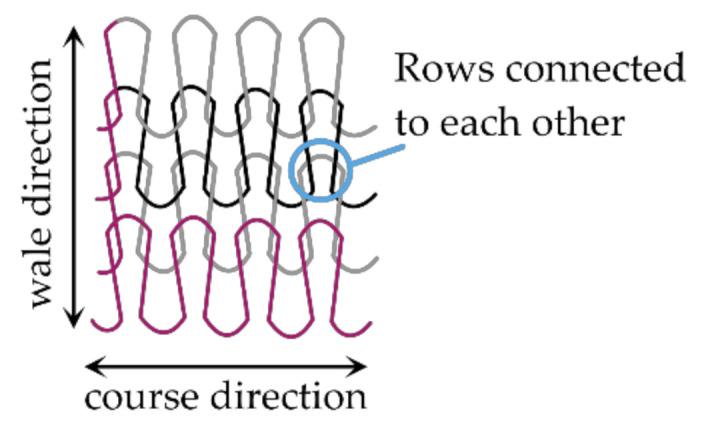
Knit structure consisting of interlinked rows of loops; current paths indicated in pink.

Textile electrodes are a type of flexible electrode and can be fabricated by various means, of which one option is knitting with conductive yarns [24,25,26]. Knitting refers to the fabrication of a fabric structure consisting of loops with one row of loops constructed with one continuous yarn (i.e., it is not cut within a row) and each row of loops being connected to the row above and below, see Figure 1. Thereby, an area of connected conductive yarns is realized and electrical current can flow within a yarn as well as from yarn to yarn [23]. When using knitting as manufacturing technique, there is the opportunity to seamlessly integrate electrodes into a garment to create wearables, which means that only one production step is required and almost no waste is produced [27].

An important property of knitted fabrics is their permeability and flexibility arising from the loop structure [28,29]. The loops can get stretched both vertically and horizontally, and the yarns are free to move within the structure, leading to a low shear and bending modulus [18]. By this, knitted electrodes can easily adjust to the curvature of the human body while still being comfortable to the skin, and intimate skin–electrode contact is enabled [9]. This makes knitted fabrics a suitable candidate for the fabrication of wearable healthcare products, providing advantages for user comfort in terms of freedom of movement [30,31].

Textile electrodes can be used in wet or dry condition. The former can be achieved using, e.g., tap water, saline solution, or an electrode cream or gel [25,32,33,34]. Differences between dry and wet textile electrodes are present with regard to some performance aspects. Depending on the planned application, dry electrodes were often found inferior to wet textile electrodes in terms of their electrical performance. In electrotherapy, dry textile electrodes were found to not work as reliably as wet ones and were leading to higher pain levels [31]. In signal monitoring, dry textile electrodes introduce higher, unstable skin–electrode impedances, which might lead to noise and a reduced signal quality [35]. The major drawback of wet textile electrodes is the drying-out of the electrode, which is changing the electrical properties and impairing the performance [17,36]. For dry textile electrodes, on the other hand, no clear consensus exists regarding their electrical performance. Some researchers have successfully proven that textile electrodes can also be used dry [8,35,37], while others prefer the use of textile electrodes in wet condition [6,24,25,26,31,32,33,34]. Nevertheless, dry electrodes are expected to provide advantages during usage; for example, a better user compliance is enabled when no re-wetting is required, the user comfort is improved when omitting the need for an electrolyte, and problems due to drying-out are avoided. For these reasons, often a compromise between comfort and electrical performance is required when choosing the planned electrode condition.

Most research in the area of textile electrodes, both for biosignal monitoring as well as for the use in electrotherapy, has so far focused on investigating the suitability of textile electrodes as alternatives to conventional electrodes, while only a few studies systematically investigated the influence of different electrode constructions on the electrode performance. A first attempt was made by Rattfalt et al. (2007), who compared three textile electrodes fabricated from different materials and by different textile manufacturing techniques, concluding that the electrode performance depended on the manufacturing technique [23]. Another approach was made by Paiva et al. (2015), who compared different knitted electrodes to find the “optimum” construction in terms of electrode performance for signal monitoring with a focus on surface smoothness and choice of conductive yarn. As a practical approach, they used a series of characterization methods starting with a big sample number and narrowing it down by excluding the worst performing electrodes after every test [38]. By this, the authors determined the best suitable electrode construction for EMG electrodes out of the compared knitted structures. However, no systematic analysis of the influence of specific knitting parameters could be found in literature. Hence, a need was detected to assess the effect of distinct construction parameters on the electrode performance.

Opportunities are seen to improve the performance of textile electrodes by applying pressure to the electrode to enhance the skin–electrode contact. A first investigation was performed by Beckmann et al. (2010), who compared different textile electrodes on a skin dummy setup, finding that a contact force improved the contact impedance [39]. Therefore, this approach was included in the present investigation to assess the influence of pressure on the electrode performance in combination with different electrode constructions.

The main aim of this work was to find indications for how specific electrode construction parameters (size, shape, density, topography) in combination with the external parameters electrode condition (i.e., dry or wet) and applied pressure influence the resulting contact impedance of the system, namely the skin–electrode impedance, referred to as three-electrode contact impedance, and the dummy-electrode impedance, i.e., the two-electrode contact impedance. As a result of this, the contact impedance-influencing factors should be determined, and recommendations for how to reduce the system’s impedance should be established.

## 2. Materials and Methods

### 2.1. Fabrication of Knitted Electrodes

The electrodes, consisting of silver-plated polyamide (Ag/PA) yarns (Shieldex^®^ 177/17/1 dtex Z100 by Statex Produktions- und Vertriebs GmbH, Bremen, Germany), were seamlessly integrated into a non-conductive polyester single jersey (PET 167/32/1 dtex, three yarns in one yarn carrier) using an industrial flat knitting machine (CMS 330 TC by H. Stoll AG & Co. KG, Reutlingen, Germany). A double jersey was used for the electrode area, meaning that the face side of the fabric consists of the conductive Ag/PA yarn, while the back side is insulated with a PET knit. 

**Figure 2:**
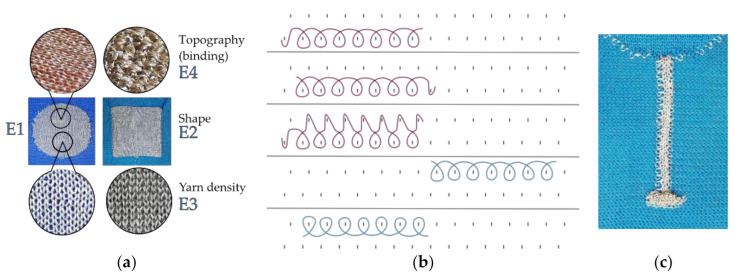
(**a**) The four different electrode versions E1–E4. (**b**) Excerpt from simplified technical drawing of knitting binding for electrode E4 with tuck stitch variation. The pink loops are made from conductive yarn to create the electrode area, while the blue yarn is non-conductive PET to knit the surrounding fabric. (**c**) Integrated textile lead with a tail in the end.

Four electrode versions were manufactured, which possess varying construction parameters, as presented in Figure 2a, with a conductive area of about 17–21 cm^2^ (usual shrinkage occurred after knitting and steaming; therefore, the areas are slightly varying between the versions).

The “comparison version”, E1, is a circle with two conductive yarns forming a plain knit binding. From this, one parameter was varied, respectively, to construct a new version, which either has a different shape (E2: square with rounded corners), a higher yarn density (E3: three conductive yarns), or a different binding to create an uneven surface topography (E4: tuck stitches integrated in plain knit, see Firgure2b). Additionally, electrode E1 was produced in three sizes (i.e., 17.4, 26.1, and 35.6 cm^2^). Therefore, six different electrode samples were knitted. A textile lead (5 × 0.5 cm) was included on the back side of the samples, as shown in Figure 2c, which is connecting the electrode area to a “tail” (0.5 × 1 cm, standing out of the fabric surface), where instrumentation can be attached.

### 2.2. Impedance Testing Methods

The electrical characterization of the electrodes was divided into two separate test series. In Series I, the skin–electrode impedance, in the following referred to as three-electrode contact impedance, was measured on a human arm, similar to the setup used by Yun-Hsuan et al. (2014), while in Series II, the pressure-dependent impedance, in the following referred to as two-electrode contact impedance, was assessed on an agar dummy similar to the study by Beckmann et al. (2010) [39,40]. In both series, an electrical impedance spectroscope (PGSTAT 204 with FRA 32M module, Metrohm Autolab, Utrecht, Netherlands) was used in potentiostatic mode to measure the impedances of the respective system. A sinusoidal voltage (amplitude 0.01 V) was applied, and the frequency-dependent impedance was automatically calculated from the measurement data using a frequency scan from 1 MHz down to 0.1 Hz with 10 points per decade.

**Figure 3:**
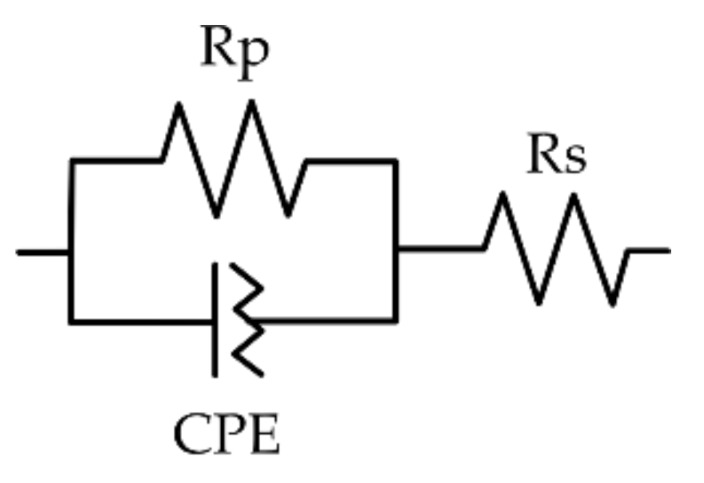
Equivalent circuit to represent the contact impedance of textile electrodes.

For the analysis, besides comparing the measured impedance values, equivalent circuits were calculated for the experimental systems. For the modelling, the integrated tool in the EIS software NOVA 2.4.1 (Metrohm Autolab, Utrecht, Netherlands) was used. As suggested by Zhou et al. (2015), to represent the skin–electrode system, a basic circuit consisting of a parallel resistor Rp and constant phase element CPE in series with another resistor Rs was chosen, see Figure 3 [31]. Within this, Rs represents the total resistance of electrode, wires, and the body/agar; Rp was used to model the charge transfer resistance; and the CPE represents the double-layer capacitance, according to following equation:

$$Z_{CPE}=\frac{1}{Y_{0}{\left(j\omega \right)}^{N}}$$

where *Y*_0_ corresponds to the admittance of an ideal capacitance and *N* is an empirical constant that can be located between 0 to 1. For the circuit fitting, a maximum number of 300 iterations was chosen with a maximum of 50 iterations without improvement. Further, a maximum change of 0.001 in goodness of fit χ^2^ (scaled) was used, and each data point was multiplied by a weight factor, i.e., the inverse of the square of the impedance modulus.

#### 2.2.1. Series I: Influence of Electrode Construction 

**Figure 4:**
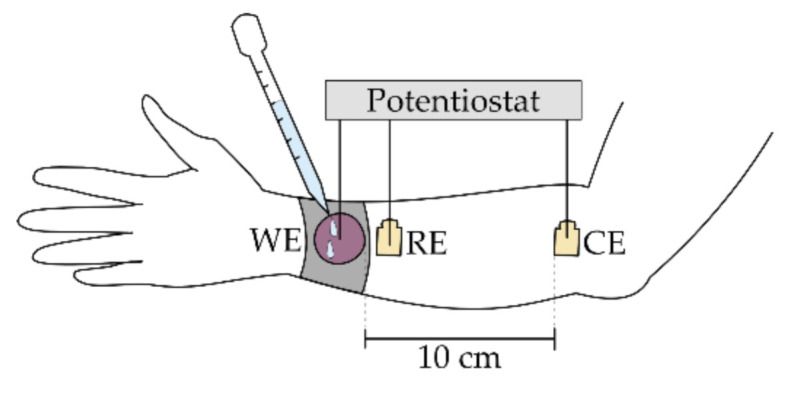
Measurement setup on human forearm (Series I) with the textile electrode indicated in pink and the conventional electrodes indicated in yellow.

In Series I, the different electrode versions were characterized regarding their influence on the impedance behavior of the system when applied to a human forearm using a three-electrode configuration with the textile electrode as working electrode (WE) and conventional, self-adhesive Ag/AgCl electrodes (23 × 34 mm, Fiab SpA, Firenze, Italy) as counter electrode (CE) and reference electrode (RE). The analyzed system consisted of the impedances of the textile electrode, of the skin and body tissue, as well as of the skin–electrode interface, and the measured three-electrode contact impedance is the combination of those individual impedances [40]. The electrode positions were kept constant for all measurements performed in Series I with a distance of 10 cm between WE and CE and a distance of 1 cm between WE and RE, as shown in Figure 4. To reduce variations arising from individual differences in body impedance, all measurements were performed on one subject only (female, 25 years old). 

One testing cycle included three impedance measurements performed in a row without re-attaching the electrodes. Five replicates of this testing cycle were made per electrode sample and per electrode condition, i.e., dry or wetted with tap water (1 mL/20 cm^2^), where each replicate was performed on a different day. Tap water was chosen as electrolyte, because advantages were seen regarding the user convenience for future applications in terms of its easy accessibility as well as the possibility of applying it from the back side of the electrode, i.e., the outside of a garment when integrated into a wearable. Thus, in future applications, the electrode can be wetted and re-wetted without taking off the garment. Additionally, compared to when using other electrolytes, no washing of the electrode is required after use.

#### 2.2.2. Series II: Influence of Pressure

**Figure 5:**
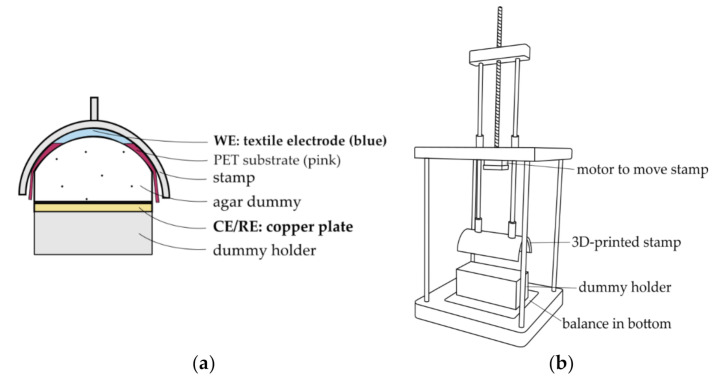
(**a**) Side-view of the dummy setup in Series II. (**b**) Testing rig for Series II.

In Series II, the influence of pressure application to the electrode on the two-electrode contact impedance was investigated. To keep the contact force controlled and uniform, a water-based agar dummy (200 mL deionized water and 7.5 g of high gel-strength agar, Sigma Aldrich A9799, St. Louis, MO, USA) was chosen instead of a human subject. The impedance was measured in a two-electrode setup with the textile electrode as WE, placed on top of the dummy, and a copper plate placed below the dummy as CE/RE (see Figure 5a). Thus, the measured two-electrode contact impedance consisted of the impedances of WE, CE/RE, the dummy impedance, and the interface impedances of the dummy and the electrodes [39]. Potential reaction products occurring at the surface of the CE/RE were removed by sanding the copper plate after every four hours of testing. The dummy was placed in a custom-built testing rig, shown in Figure 5b, which has a 3D printed stamp to apply a controlled pressure to the agar-electrode setup. A new dummy was used for every day of testing, as the dummy is expected to dry out over a longer period of time, thereby changing its electrical and mechanical properties.

To perform one testing cycle, the stamp gave a pre-force of 200 g to the testing setup, followed by three impedance measurements; then, the pressure was increased to the investigated pressure level (indicated by the force at 400, 600, and 1000 g), and again, three measurements were performed. Eight replicates of the testing cycle were performed per electrode sample and pressure level. Only electrodes with the approximate same size were evaluated in this experimental series to ensure a comparable pressure distribution for all tested electrodes. Therefore, the two bigger sizes of electrode construction E1 were excluded.

## 3. Results 

### 3.1. Series I: Influence of Electrode Size and Construction in Wet and Dry Condition

Series I was aiming to investigate the influence of electrode size and construction on the three-electrode contact impedance in dry and wet condition. The contact impedances were measured over the entire frequency range, and all measured frequencies were included in the analysis. The contact impedances at high frequencies showed smaller variations than at low frequencies, but the comparison of the different electrode versions showed the same trends for the respective parameter influences at all frequencies. Therefore, in the following, only the contact impedances for 39 Hz are presented, as this is a commonly used frequency in electrotherapy [20,41,42,43].

Regarding the modelled equivalent circuits, the total resistance in the low frequency range is the sum of Rs and Rp. In the present study, Rs was calculated to be located in a range of 60–70 Ω on the agar dummy and 80–100 Ω on a human arm, which aligns with findings by Zhou et al. (2015), who found similar Rs values with around 100 Ω for wet and dry textile electrodes on a human leg [31]. This is very low compared to the calculated charge transfer resistances Rp, which are having magnitudes in the order of 10^4^ Ω in wet and 10^8^ Ω in dry condition. Therefore, Rs can be neglected for the low frequency range in which the planned applications, such as electrostimulation and signal monitoring, are located. Therefore, the calculated Rs values are not presented in the following. 

#### 3.1.1. Influence of Size

**Figure 6:**
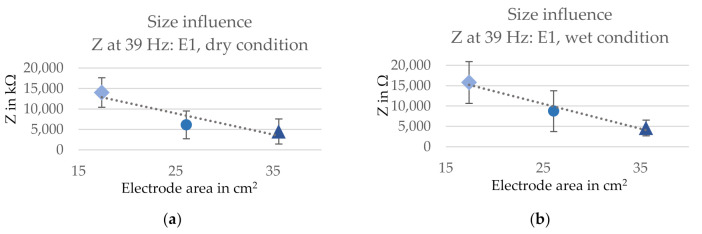
Influence of the size on the three-electrode contact impedances for electrode version E1 at 39 Hz in (**a**) dry and (**b**) wet condition. SD indicated with error bars and linear trend line as dashed line.

The influence of the electrode size (i.e., the conductive area) on the three-electrode contact impedance is presented in Figure 6 for electrode E1 in three sizes. The impedance decreased for a bigger electrode area in both dry (from 14 to 4.5 MΩ) and wet (from 15.8 to 4.6 kΩ) condition, showing an inversely proportional relation between contact impedance and electrode area in wet condition. In dry condition, on the other hand, the impedance decrease slightly deviated from a fully linear manner. However, this might be attributed to the experimental setup. The skin and body tissue impedance is varying between body locations. If an electrode has a bigger area, a different part of the arm is in contact with the electrode. Therefore, the three-electrode contact impedance is influenced by these variations. Additionally, in wet condition, the electrode slightly sticks to the skin. Thus, the effective contact area is matching the electrode area more precisely, as the entire electrode is in close contact with the skin. In dry condition, the electrode contact might not have been as good. Hence, the effective contact area is subject to variations between measurements, particularly in dry condition, which affects the measured three-electrode contact impedances.

**Figure 7:**
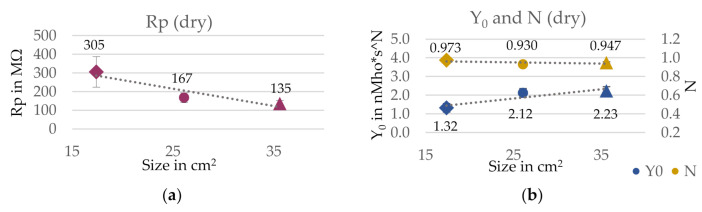

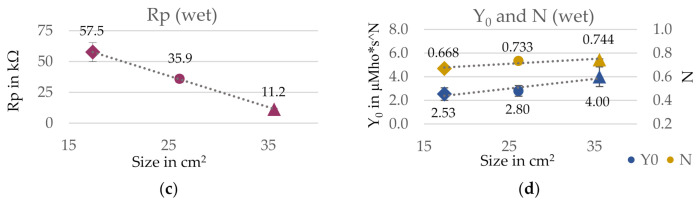
Influence of the electrode size on the equivalent circuit elements for electrode version E1. (**a**) Charge transfer resistance Rp and (**b**) CPE parameters Y_0_ and N in dry condition. (**c**) Charge transfer resistance Rp and (**d**) CPE parameters Y0 and N in wet condition. Estimated error indicated with error bars and linear trend lines as dashed lines. Goodness of fit in dry: χ^2^ (17.4 cm^2^) = 16.9; χ^2^ (26.1 cm^2^) = 9.4; χ^2^ (35.6 cm^2^) = 7.6; and in wet: χ^2^ (17.4 cm^2^) = 8.4; χ^2^ (26.1 cm^2^) = 4.8; χ^2^ (35.6 cm^2^) = 5.2.

The results for the equivalent circuit are presented in Figure 7 for wet and dry condition. Linear relations could be found for the electrode size and the calculated circuit elements. The charge transfer resistance Rp showed a linear decrease both in wet and dry condition for an increase in electrode size. This aligns with the analysis of the impedance, which showed the same trend. Further, as expected, Rp is significantly higher in dry (135–305 MΩ) than in wet condition (11.2–57.5 kΩ) due to the lack of electrolyte in the interface. For the capacitance Y_0_, an increase could be observed upon an increase in electrode size for both conditions, while the empirical constant N was only very slightly affected by the electrode size, especially in dry condition. This implies that a bigger electrode area is favorable, as a higher Y_0_ and a bigger N are to be preferred to reach a more ideal capacitive behavior. However, it must be noted that the goodness of fit χ^2^ was extraordinarily high for all electrode sizes, which means that the reliability of the circuit model is limited.

#### 3.1.2. Influence of Electrode Construction Parameters

**Figure 8:**
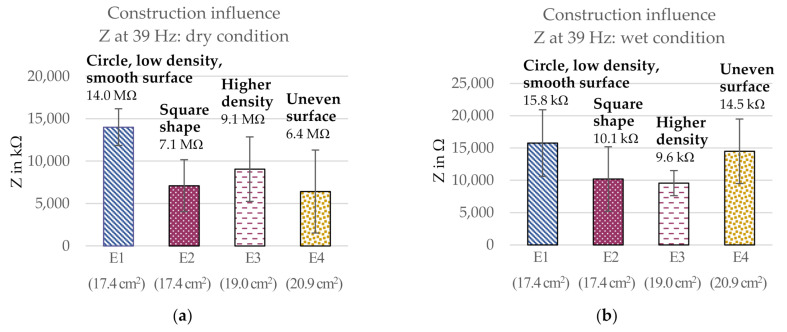
Influence of the electrode construction on the three-electrode contact impedance at 39 Hz in (**a**) dry condition and (**b**) wet condition. SD indicated with error bars.

The influence of the investigated construction parameters on the three-electrode contact impedance is shown in Figure 8. The electrode areas are indicated in the graphs, as they were varying slightly. The comparison shows that the electrode with a smooth surface, lower yarn density, and a circular shape (E1) gave the highest three-electrode contact impedance both in dry (14 MΩ) and wet (15.8 kΩ) condition. Adding water as an electrolyte greatly reduced the contact impedance for all electrodes, with the overall ranking of the electrodes remaining similar, albeit minor changes taking place. While electrodes E1–E3 behaved similarly to each other in dry and wet condition, the behavior of electrode E4 (uneven surface) was changed significantly when adding water as an electrolyte. The three-electrode contact impedance was increased compared to the other electrode versions, now having the second highest impedance (14.5 kΩ), compared to having the lowest impedance in dry condition (6.4 MΩ). Further, the contact impedance of E3 (higher density) was reduced compared to the other electrodes now having a similar impedance as E2 (square electrode) in wet condition with around 10 kΩ. 

**Figure 9:**
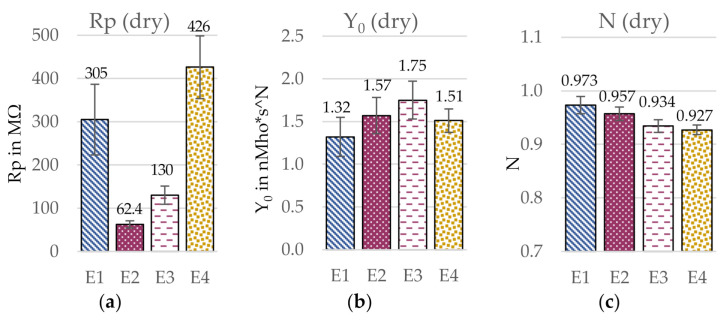
Influence of the electrode construction on the simulated circuit elements. (**a**) Charge transfer resistance Rp, (**b**) CPE admittance Y_0_, and (**c**) empirical constant N in dry condition. Estimated error indicated with error bars. Goodness of fit χ^2^ (E1) = 16.9; χ^2^ (E2) = 10.5; χ^2^ (E3) = 10.2; χ^2^ (E4) = 8.1.

**Figure 10:**
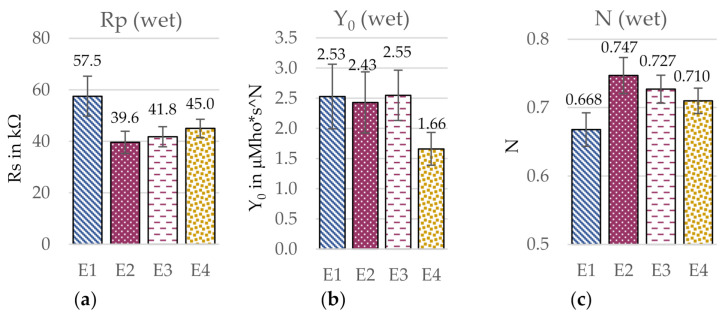
Influence of the electrode construction on the simulated circuit elements. (**a**) Charge transfer resistance Rp, (**b**) CPE admittance Y_0_, and (**c**) empirical constant N in wet condition. Estimated error indicated with error bars. Goodness of fit χ^2^ (E1) = 8.4; χ^2^ (E2) = 7.4; χ^2^ (E3) = 5.3; χ^2^ (E4) = 4.8.

To compare the different electrodes in a more quantitative way, a circuit simulation was performed. The respective equivalent circuit elements are presented in Figure 9 and Figure 10. For the comparison of the charge transfer resistances Rp in dry condition, the electrode with the uneven surface (E4) showed by far the highest Rp with 426 MΩ, while in wet condition, the circular electrode with a low yarn density and smooth surface (E1) had the highest Rp with 57.5 kΩ. Further, differences between Rp in wet condition were much smaller than in dry condition, which presumably results from the construction having a reduced influence as effect of the presence of water, which reduces Rp significantly compared to the dry electrodes. In dry condition, the capacitance Y_0_ was highest for the electrode with a high yarn density (E3) with 1.75 nMho*s^N and lowest for the circular electrode with the low yarn density (E1 with 1.32 nMho*s^N), but the values were generally similar. In wet condition, on the other hand, the electrode with an uneven surface (E4) led to a significantly lower Y_0_ with 1.66 μMho*s^N compared the other electrode constructions, which in turn showed rather similar values around 2.5 μMho*s^N. Regarding the calculated N values, differences between the electrode constructions were rather small, especially in dry condition. In wet condition, the circular electrode with a smooth surface and a low yarn density (E1) led to the lowest N value with 0.668, while the other electrode constructions showed rather similar values between 0.71–0.75. However, it must be noted that the goodness of fit χ^2^ was extraordinarily high for all electrode versions, wherefore the results from the EC are limited in certainty. In the following, the individual construction parameters will be analyzed more in detail.

**Figure 11:**
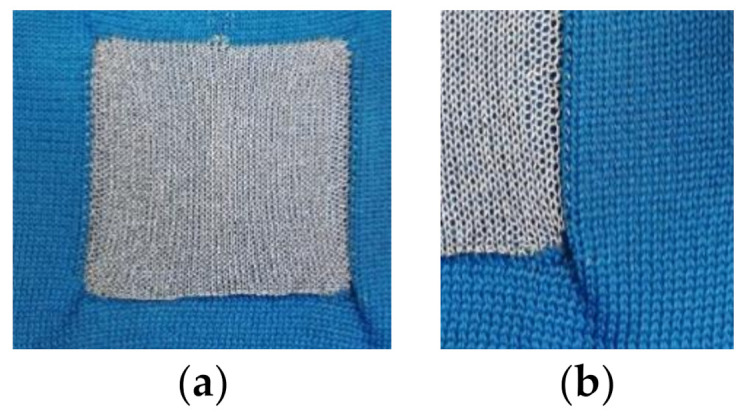
(**a**,**b**) Rolling in of side edges for the square shaped electrode version E2 on the face side.

The analysis of electrode version E2 with a square shape showed a problem related to the knitting construction. The non-conductive fabric around the electrode tends to form creases at the corners, as shown in Figure 11. As a result, the edges slightly roll over, thereby partly covering, i.e., potentially insulating, the edge area of the electrode when the fabric is not sufficiently stretched. Because of this, the skin contact might have been impaired in the edge areas, thus reducing the effective contact area. This suggests that an optimization of the knitting construction of the square-shaped electrode E2 to avoid the rolling tendency might further reduce the contact impedance by increasing the effective contact area. 

**Figure 12:**
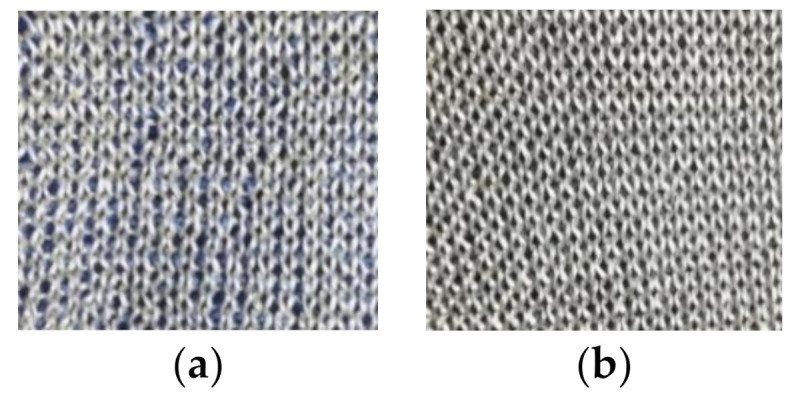
(**a**) Close-up of electrode construction with low yarn density (E1). (**b**) Close-up of electrode construction with high yarn density (E3).

A higher yarn density of conductive yarns seemed to decrease the three-electrode contact impedance with an impedance difference of 5.0 MΩ at 39 Hz (corresponding to a decrease of 35.3%) in dry condition and 6.2 kΩ (decrease of 39.4%) in wet condition. The analysis of the EC supported this trend, as the charge transfer resistance Rp of electrode E3 (higher yarn density) was lower both in wet and dry condition than the Rp of electrode E1 (low yarn density). However, the size difference between the two electrode versions E1 and E3 in the density comparison must be considered. Electrode E1 with a lower yarn density has a slightly smaller area than electrode E3 with a higher yarn density (1.6 cm^2^ difference in areas). As found in the previous size comparison, a larger electrode leads to a reduced contact impedance and lower charge transfer resistance Rp. Thus, the three-electrode contact impedance and the Rp of electrode E3 (higher density) are expected to slightly increase when the size is reduced to match electrode E1. In this case, the impedance difference between the two electrodes would be reduced. Therefore, the found influence of a higher yarn density cannot be considered as significant, as the size influence cannot be excluded with certainty. Thus, the observation that a higher density reduced the contact impedance is considered an indication only. Nevertheless, the potential trend can be explained as follows. A higher yarn density means that the electrode area is more densely covered with conductive yarns. Therefore, the full electrode area is available as contact area, while for a lower density, gaps are visible between the yarns, see Figure 12, which means a difference between electrode area and effective contact area is expected. This suggests that a higher yarn density leads to a better skin contact and thereby improves the electrode performance. This is supported by the EC analysis in which the electrode with a higher yarn density (E3) showed the highest capacitance Y_0_ in both conditions, which suggests an improved skin–electrode contact for this electrode construction.

**Figure 13:**
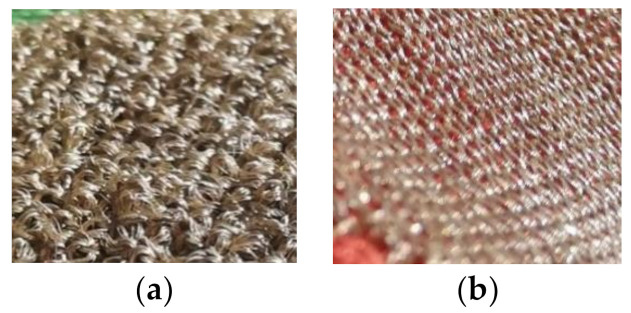
(**a**) Close-up of electrode version with an uneven surface (E4). (**b**) Close-up of electrode version with a smooth surface (E1).

Additionally, for the analysis of the topography influence on the three-electrode contact impedance, a size difference between the two compared electrodes must be considered, with electrode E4 (uneven surface) having a bigger area. Here, a difference of 3.5 cm^2^ was present, which is expected to have affected the contact impedance significantly. If the assumption for an inverse size-impedance relation holds true for electrode version E4 (uneven surface), the contact impedance would increase when reducing the electrode size. Thus, the observation that an uneven surface reduces the contact impedance can only be considered an indication, as the certainty is limited. However, a possible explanation for the observed trend is that skin irregularities can be compensated by the loops that are standing out of the surface, see Figure 13. Thus, the skin contact is improved and the three-electrode contact impedance in turn reduced, which is supported by a higher capacitance Y_0_ for electrode E4 (uneven surface) than for electrode E1 (smooth surface) in dry condition. In wet condition, on the other hand, an opposite trend was observed. Both the electrode with a smooth surface as well as the electrode with an uneven surface showed approximately the same contact impedance. In this case, when the size of electrode E4 (uneven surface) is reduced, the impedance would increase, thus leading to a higher contact impedance compared to E1 (smooth surface). Therefore, here, an indication was found that a loop structure increases the contact impedance in wet condition. This can be explained by skin irregularities being compensated by the water instead of by the electrode structure, as fluids have a better capability to adjust to the uneven skin than textiles. In turn, for the electrode with the loop structure, the water distribution within the interface of skin and electrode is supposedly more irregular, including disturbances arising from the loops. The applied water cannot form a closed film between electrode and skin, as it gets trapped in the loops. Additionally, air might also get trapped inside the structure, thereby greatly increasing the local impedance. Thus, the electrolyte does not manage to create a homogeneous interface, and the contact impedance is increased compared to a smooth surface. This is supported by the findings from the EC where a significantly low capacitance Y_0_ was found for the electrode with an uneven surface (E4) in wet condition, as a low Y_0_, i.e., non-ideal capacitive behavior, is caused by a lack of homogeneity of the interface, leading to an impaired skin–electrode contact. Therefore, when wet electrodes are being developed, a smooth surface seems preferable over a rough surface composed of loops in regard to the contact impedance.

Differences between the four electrode constructions became visible when comparing the variation in three-electrode contact impedance data. Electrode E4, which has an uneven surface, shows the biggest standard deviations (SD), with 4.9 MΩ (dry) and 8.7 kΩ (wet), while the other three constructions show similar magnitudes of SD to each other in the range of 3.0 MΩ (dry) and 5 kΩ (wet). This means that an uneven electrode surface leads to a less stable contact impedance, which can supposedly be related to the variations in quality of skin contact arising from the loops. The loops are rather long so that not the entire electrode area is touching the skin as long as the electrode is not actively pressed to the arm. Accordingly, the contact area is relying on the tops of the loops, which are touching the skin, rather than on the entire conductive area of the fabric. Therefore, the contact area is harder to estimate or control. These deviations showed a visible influence on the electrode performance, and accordingly, a smoother surface is to be preferred when a stable contact impedance is required.

### 3.2. Series II: Influence of Pressure Application and Electrode Construction

**Figure 14:**
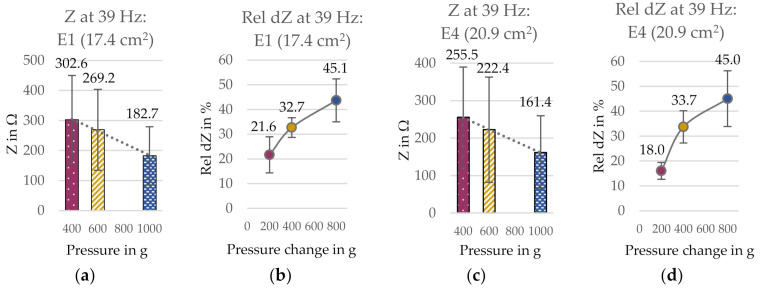
(**a**) Contact impedance Z and (**b**) relative impedance change rel dZ at 39 Hz for electrode E1 (smooth surface, circle, low yarn density). (**c**) Contact impedance Z and (**d**) relative impedance change rel dZ for electrode E4 (uneven surface). SD pictured as error bars.

Series II was aiming to determine the influence of pressure application on the dummy-electrode impedance, i.e., the two-electrode contact impedance, in combination with the electrode construction. The results for electrodes E1 and E4 are exemplarily presented in Figure 14 at 39 Hz. The relative impedance change rel dZ is the magnitude of contact impedance change upon pressure change (i.e., from the pre-force to the investigated pressure level), calculated in percent. Thus, the value is always positive and not indicating the direction of change. The contact impedance Z was reduced upon a higher pressure application, indicated by the applied force, based on an inversely linear relation for all four electrodes. Within this, the relative impedance change was bigger for a bigger pressure change, though not fully linear. The curve is steeper in the beginning and gets flatter for the bigger pressure changes for all electrodes. This suggests that a limit in form of a maximum impedance change was being approached, and therefore, the contact impedance change did not behave linearly proportional to the pressure change. Nevertheless, this limit was not reached in the performed experiments.

**Figure 15:**
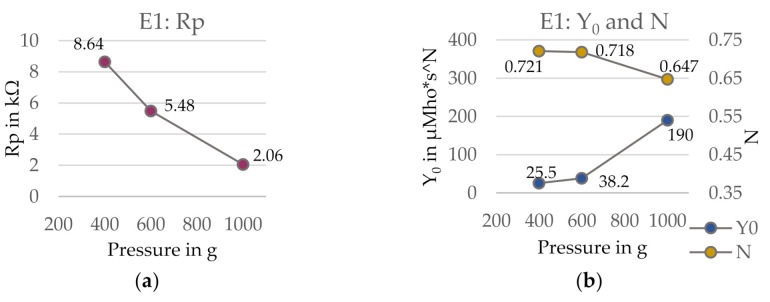

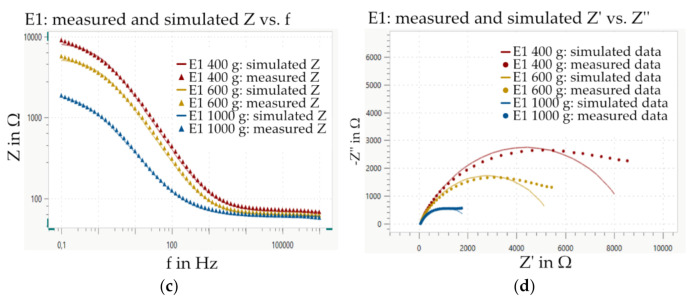
(**a**,**b**) Circuit elements Rp and CPE parameters Y_0_ and N for electrode E1 on agar dummy with pressure applied to the system. Estimated errors are indicated by error bars, which are not visible because they are too small. (**c**,**d**) Comparison of measured data and simulated EC curves over the entire frequency range for electrode E1 (circle, low yarn density, smooth surface). Goodness of fit χ^2^ (400 g) = 0.23; χ^2^ (600 g) = 0.21; χ^2^ (1000 g) = 0.08.

The modelled circuit elements for electrode version E1 (circle, high yarn density, smooth surface) are presented in Figure 15a,b. The charge transfer resistance Rp and the empirical constant N decreased for an increase in pressure while the capacitance Y_0_ increased, although the trends were not purely linear. The estimated errors were found to be very small with maximum 3.5%, which suggests that the equivalent circuit used for modelling is valid for the investigated system. This can also be seen in the comparison of measured data and fitted and simulated EC curve in Figure 15c,d, in which the simulated curves and measured data points show a remarkable agreement regarding the impedance Z. However, it must be noted that the goodness of fit values χ^2^ are still rather high.

#### 3.2.1. Shape and Pressure

**Figure 16:**
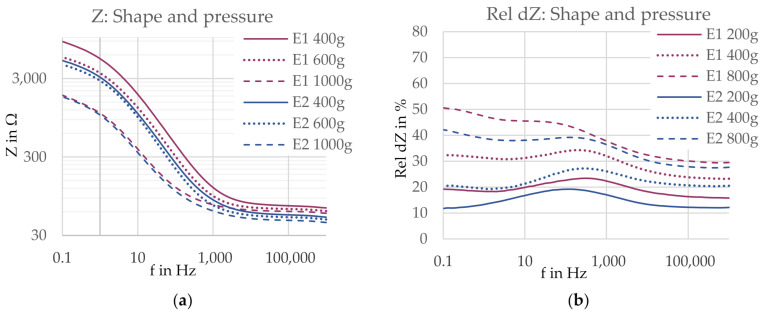
(**a**) Contact impedance Z and (**b**) impedance change rel dZ for electrodes with a circular shape (E1, 17.4 cm^2^, pink) and a rectangular shape (E2, 17.4 cm^2^, blue). Electrode versions are marked with different colors, and pressure levels are marked with different line styles.

The comparison of the pressure-dependent contact impedances for the circular electrode (E1) and the square-shaped electrode (E2) are shown in Figure 16a for the entire frequency range. The electrode shape is indicated by the line color and the applied pressure is represented by the line style. When comparing the two electrode versions at the same pressure level (i.e., the same line style), a square-shaped electrode (E2) reduced the contact impedance Z compared to a circular electrode (E1) in the high frequency range (f > 1 kHz). For the lower frequencies, however, no clear influence of the shape was visible. At the pressure level of 400 g, the square electrode showed a significantly lower contact impedance, but at 600 and 1000 g, respectively, the contact impedances for both electrodes were similar. Thus, when applying a pressure of more than 400 g, the influence of pressure was pronounced in the low frequency range, while the influence of the electrode shape was dominant in the higher frequencies for all pressure levels. The factor weight of these two factors changes at a frequency of around 1 kHz.

For the relative impedance change rel dZ, see Figure 16b, the square-shaped electrode showed a lower impedance change at all pressure levels compared to the circular electrode over the entire frequency range. Nevertheless, the influence of pressure is pronounced over the influence of the shape, as the curves of the same pressure level are grouped together, particularly in the high frequency range where the differences between the respective pressure levels are bigger than between the two electrode versions at the same pressure level.

#### 3.2.2. Density and Pressure

**Figure 17:**
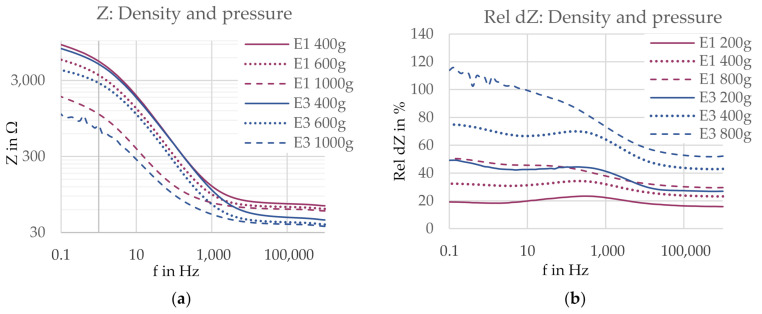
(**a**) Contact impedance Z and (**b**) impedance change rel dZ for electrodes with a lower yarn density (E1, 17.4 cm^2^, pink) and higher yarn density (E3, 19.0 cm^2^, blue). Electrode versions are marked with different colors, and pressure levels are marked with different line styles.

A higher yarn density (E3) reduced the contact impedance at all pressure levels, as visible in Figure 17a, with the exception of the frequency range between 10 Hz and 1 kHz for a pressure level of 400 g, where the impedance curves of the two electrodes meet. In the low frequency range, the curves are forming “pressure groups”, as the curves of the same pressure level of both electrode versions show similar contact impedances, while there is a clearer difference between the curves of different pressure levels. In high frequencies, however, this behavior changes. Here, the impedance curves of the electrode versions drift apart, thereby forming “version groups”. The critical frequency where the behavior is changing is located around 1 kHz. This means the factor weight of pressure is pronounced for low frequencies, while the factor weight of the yarn density is dominating in higher frequencies. 

The two compared electrodes possess a size difference of 1.6 cm^2^, which might affect the contact impedance. As found in Series I, the three-electrode contact impedance measured on a human forearm is inversely related to the electrode area. If this hypothesis holds true in the present experimental setup for the contact impedances of both electrodes measured on an agar dummy, the impedance of electrode E3 (higher density) is expected to slightly increase if the size is reduced. Thus, again, the contact impedance difference between the two electrodes will be reduced and therefore, the present observations are only considered indications.

For the comparison of the relative impedance change rel dZ, shown in Figure 17b, the electrode with a higher yarn density (E3) showed a bigger impedance change at all pressure levels. The influence of the yarn density is clearly pronounced over the influence of the applied pressure.

#### 3.2.3. Topography and Pressure

**Figure 18:**
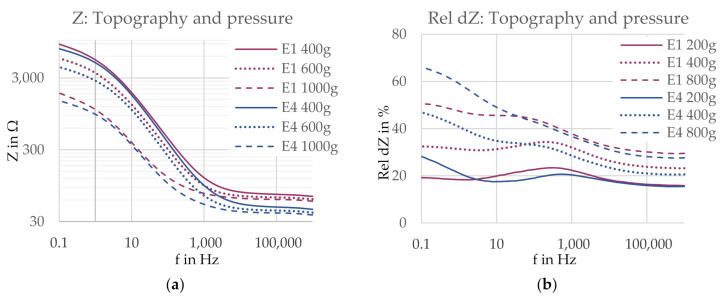
(**a**) Contact impedance Z and (**b**) impedance change rel dZ for electrodes with a smooth surface (E1, 17.4 cm^2^, pink) and an uneven surface (E4, 20.9 cm^2^, blue). Electrode versions are marked with different colors and pressure levels are marked with different line styles.

The analysis of the influence of the topography in combination with pressure, presented in Figure 18a, shows that also in this comparison, the influence of pressure is pronounced in the low frequency range, while the influence of the electrode construction, in this case the surface structure, is dominant in the high frequency range, with a critical frequency located around 1 kHz. At high frequencies, the uneven surface reduced the contact impedance at all pressure levels. At lower frequencies, only slight to no differences could be observed between the contact impedances of the two electrodes at the same pressure level. 

A size difference of 3.5 cm^2^ is present between the two electrodes. Assuming the previously found inverse size–impedance relation, the contact impedance difference between the two electrodes is reduced once the electrodes have the same area. Accordingly, again the observations are considered indications, which must be confirmed by excluding the size influence. 

The comparison of the relative impedance change rel dZ in Figure 18b shows that the impedance change was rather similar for both electrodes at the same pressure level except for at frequencies below 10 Hz. Here, an uneven surface led to a bigger relative impedance change. At higher frequencies, the pressure change is the dominating factor determining the magnitude of impedance change, while the influence of the surface structure is greatly reduced. 

## 4. Discussion

The main aim of this research was to find indications on how the electrode construction parameters influence the resulting contact impedance to provide guidelines for choosing a favored electrode construction for knitted electrodes. A “good” electrode performance requires the skin–electrode system to have a low impedance as well as low variation in impedance [23].

### 4.1. Factors Lowering the Contact Impedance

As already found, for the equivalent circuit in the low frequency range, the series resistance Rs can be neglected, as the biggest contribution to the overall contact impedance can be ascribed to the charge transfer resistance Rp. Because electrostimulation and signal monitoring are performed using low frequencies, this implies that, to reduce the contact impedance, aiming to reduce Rp is advisable. Further, regarding the CPE, a higher capacitance Y_0_ and a larger empirical constant N are preferred to reduce the non-ideal capacitive behavior.

#### 4.1.1. Electrode Condition

In the performed experiments, the dominant factor determining the contact impedance was the electrode condition, which was also supported by the findings from the equivalent circuits. Wet textile electrodes are known to have a greatly reduced impedance compared to dry electrodes, as found in various earlier studies for example by Zhou et al. (2015) or Márquez Ruiz (2013) [31,44]. This observation was confirmed in the present study. When adding water, the charge transfer resistance Rp was significantly reduced, while the capacitance Y_0_ was increased. Regarding the impedance behavior, the electrode construction comparison mostly led to the same results in wet and dry condition in terms of favored construction parameters, which implies that adding water does not majorly affect the electrical behavior as such, but instead only lowers the contact impedance in the same way for most electrode constructions. However, one exception was found for an electrode with an uneven surface consisting of loops. In this case, in wet condition the electrode performance was worsened compared to the other electrode constructions, which suggests that here, the electrode behavior was noticeably modified by the presence of water as electrolyte. Therefore, the planned condition must be considered when choosing a suitable electrode construction. 

Additionally, minor aspects improving the performance of wet electrodes compared to dry electrodes are found in the behavior of wetted textiles. A wet fabric tends to slightly stick to the skin, thereby being less prone to being influenced by body movements. Further, the skin–electrode contact is improved when small skin irregularities are compensated by the water, as a fluid can adjust to the skin more easily than a textile. Despite those advantages of wet electrodes, the biggest drawback is the occurrence of drying-out impairing the electrode performance over time. This could not be observed in the present study, as the testing cycle was too short to be able to observe a significant effect. Nevertheless, for a longer time span, the contact impedance is expected to increase resulting from the reduced wetness of the electrode. First approaches to improve the moisture retention or to integrate a controllable re-wetting mechanism can already be found in literature. For example, Weder et al. developed a water reservoir for embroidered electrodes to achieve re-wetting based on water vapor [45]. Nevertheless, more research is required to solve the drying-out problem.

Moreover, another problem observed for wet electrodes was a discomfort related to the “wet feeling” when the skin is in constant contact with the water. Especially for a longer time duration, this led to a cold feeling on the skin, and the wetness itself might be perceived as uncomfortable. To reduce these problems, the water amount should be kept as low as possible, which in turn is oppositional to the aim of keeping the electrode wet over the entire treatment time. Hence, these aspects limit the suitability of wet textile electrodes to applications that only require short usage durations of maximum a few hours per session. It seems advisable to perform research on which water amount is most suitable, compromising both aspects, as well as how to control the present water amount in a precise way.

#### 4.1.2. Electrode Size

The skin–electrode impedance and the contact area of electrode and skin are known to be inversely related [40]. Thus, when assuming the electrode area to be equal to the contact area, the contact impedance is expected to decrease proportionally with a bigger electrode size. This expectation was confirmed for the electrode construction investigated in this study, which had sizes between 17.4 and 35.6 cm^2^, and the observations were supported by the EC simulation in which a bigger area lowered the charge transfer resistance Rp and increased the capacitance Y_0_. Hence, a bigger electrode is favorable to achieve a lower contact impedance. However, it must be considered that in electrotherapy, the stimulation selectivity is expected to be reduced when using bigger electrodes, which means the stimulation efficiency is affected [46]. Therefore, both aspects should be compromised when choosing the electrode size. For future work, investigations are needed regarding the size influence for other electrode constructions, as here only one electrode construction was tested to evaluate the size influence. However, when changing construction parameters, particularly the surface topography, the effective contact area of skin and electrode are affected, which might become visible in the influence of electrode size on the contact impedance.

#### 4.1.3. Electrode Construction

The contact impedances of different electrode constructions were measured in a frequency scan using two different testing setups; one on a human forearm and one on a water-based agar dummy. Both systems showed the same behavior regarding the influence of the electrode construction on the contact impedance. A square shape reduced the contact impedance, and indications were found for a higher yarn density and an uneven surface structure lowering the contact impedance. While this was true for the entire frequency range for measurements on a human arm without applying pressure, it could only be observed in higher frequencies (f > 1 kHz) when pressure was applied to the dummy-electrode system. Thus, future work is required to validate if this limitation for the significance of the construction influence in combination with pressure also exists when testing on a human subject instead of on a skin dummy. 

The electrode shape was expected to influence the contact impedance only when a critical frequency is exceeded, which is located in the high-frequency end of the spectrum. This expectation is arising from the differences in current distribution between rectangular and circular electrodes, with an uneven charge distribution in the corners of a rectangular electrode, compared to circular electrodes, which are thought to have a more uniform current distribution [6]. Tjelta and Sunde (2015) observed that below a critical frequency, the impedance of porous electrodes or electrodes covered with films was not affected by the current density [47]. However, in the experiments performed in this study, an influence of the shape could be observed at all frequencies in wet and dry condition when tested on a human forearm, which implies that the expected critical frequency for the influence of the electrode shape does not exist for textile electrodes when placed on the human body. As textile surfaces are rather complex, the interface of textile electrodes and the skin behaves differently than for example for hydrogel electrodes. This means that findings for other electrode types cannot directly be translated to textile electrodes. Thus, the expectations could not be confirmed, and a square shape with rounded corners was found preferable to reduce the contact impedance at all frequencies. This was supported by the findings from the EC analysis, where a square shape led to a reduced charge transfer resistance Rp for both wet and dry electrodes and a higher capacitance CPE-Y_0_ in dry condition. Nevertheless, once a contact force of 400 g or higher was applied to the system measured on an agar dummy, a critical frequency could be observed at around 1 kHz, below which the applied pressure was determining the contact impedance, while the shape only showed a clear influence above this frequency. 

In conclusion, a square shape with rounded corners is preferable over a circular electrode to reduce the contact impedance. However, the impedance decrease resulting from the shape is expected not to be noticeable in low frequency applications such as electrotherapy when higher pressures are applied to the electrode. Further, in electrotherapy, problems regarding the stimulation comfort might arise from too sharp corners, which could impair the stimulation efficiency. This must be considered when choosing a suitable electrode shape.

The skin contact was expected to be improved for a higher yarn density, as more conductive fibers are present [44]. Therefore, a higher yarn density was predicted to lower the contact impedance by reducing the resistive part and increasing the current transfer [18]. This expectation could be confirmed for wet and dry electrodes on a human forearm as well as on an agar dummy for high frequencies (f > 1 kHz) when applying different pressure levels. Therefore, electrodes with a higher yarn density are to be preferred unless pressure is applied in low frequency applications. In this case, a lower yarn density appears favorable to reduce the material cost while still achieving a comparable electrode performance. However, a limit is expected for the yarn density, below which it will show an impact on the contact impedance and impair the electrode performance even when higher pressures are applied. Further, as the results could only be considered indications, electrodes with bigger density differences and same electrode areas should be investigated in future research to increase the certainty of the observations and find the minimum yarn density required. 

The last investigated textile construction parameter was the binding to change the surface topography. In an investigation by Márquez Ruiz (2013), a rougher surface was found to improve the skin contact, because it could compensate for skin irregularities more easily, while an especially flat surface was found to result in a less uniform contact of electrode and skin [44]. Therefore, for the performed experiments, it was expected that the electrode with the smooth surface leads to a higher contact impedance. This could be partly confirmed, as an indication for the expected behavior was found when measuring on a human forearm in dry condition as well as at high frequencies when applying pressure on an agar dummy. In wet condition on a human arm, however, the observed trend behaved oppositely to the expectations, because an indication was found that a surface structure consisting of loops increases the contact impedance of textile electrodes wetted with water due to an impaired skin–electrode contact. Concluding, based on these observations, the preferable surface structure depends on the condition of the electrode. However, it must be considered that this observation might not be true for all kinds of uneven surfaces. The reason for the found trend is expected to be rooted in the loops structure. Therefore, other means for creating uneven surfaces, e.g., fibers standing out instead of loops, should be investigated in future work to be able to assess the influence of the topography on the contact impedance in wet condition more generally. 

#### 4.1.4. Pressure Application

The effect of applying pressure to a dummy-electrode system on the contact impedance was investigated to assess the influence of pressure in combination with the electrode construction. It was expected that a higher pressure reduces the contact impedance with the magnitude of impedance change depending on two aspects. Firstly, for the same electrode construction, a bigger pressure change dF was expected to lead to a bigger change in contact impedance with dZ ~ 1/dF. Secondly, the magnitude of impedance change rel dZ was expected to be dependent on the combination of pressure change and electrode construction, meaning that the combined effect of construction and pressure application determines the impedance change. These hypotheses could be confirmed in the performed experiments. The impedance change was directly related to the change in pressure, and the electrode construction showed influences on the impedance change, particularly for differences in yarn density. Therefore, the combination of applied pressure and electrode construction determined the impedance change. However, the impedance change upon pressure change was not linear, which implies that a limit in form of a maximum impedance change was being approached. Therefore, future work is needed to find the optimum pressure for reducing the contact impedance. 

The effect of pressure application on the contact impedance, measured on an agar dummy, was found to be frequency-dependent. In high frequencies with f ≥ 1 kHz, the electrode construction showed a higher factor weight than the pressure application. In lower frequencies, however, the applied pressure was found to be the impedance-determining parameter, with only little influence of the electrode construction. Accordingly, if electrodes for high-frequency applications are to be developed, besides applying pressure to the electrode, the electrode construction should be considered to reduce the contact impedance. Here, a square shape is to be preferred over a circular shape, and a high yarn density of the conductive yarn is advisable. For the topography, an uneven surface should be chosen over a smooth surface structure. However, as the threshold was found to be at around 1 kHz, this is not relevant for applications within electrotherapy, because usually significantly lower frequencies are used. Here, the application of pressure to the skin–electrode system is expected to show bigger effects on reducing the contact impedance than changing the electrode construction. An opportunity is seen to improve the performance of dry textile electrodes to be comparable to wet electrodes. This would solve the problem of drying-out of wet electrodes as well as discomfort related to a “wet feeling” on the skin. Hence, for future work, the influence of pressure on the resulting contact impedance should be tested on a human subject instead of on a dummy to confirm the observations and validate the suitability of an agar dummy to simulate a skin–electrode system. In this, the user comfort should be included, because too high pressures might lead to discomfort or even pain.

### 4.2. Factors Affecting the Contact Impedance Variation

#### 4.2.1. Experimental Considerations

Rather high variations in the contact impedance data were found in the performed experiments, especially for the lower frequency range (f < 1 kHz). This can be explained by several factors related to the experimental setups. When measuring the contact impedance by placing electrodes on the human body, variations are arising from varying skin properties, which are subject to intra- and inter-day variations, as also found by Rattfalt et al. [23]. Further, in case of dry electrodes, a stabilization time is recommended in literature to stabilize the skin–electrode interface [48]. This might reduce the variation in the measured contact impedance data for dry electrodes. However, no detailed investigation regarding the effectiveness of stabilization time for electrotherapy or biosignal monitoring applications could be found, and the stabilization times chosen in other studies are differing in a wide range from just a few minutes up to one hour [35,49,50]. Thus, future work is required regarding the optimum stabilization time for dry textile electrodes.

When measuring the contact impedance on a water-based agar dummy, varying dummy properties are causing variations between measurements, as agar gel dries out when exposed to air, thus changing its electrical impedance. Even if the dummy is stored in an airtight bag until use, drying-out could not be avoided during performance of the experiments, leading to intra- as well as inter-dummy variations. Further, rather high inter-dummy variations were noticed, even though the same recipe was used for all dummies. Therefore, possibilities for making the dummy properties more stable over time as well as how to reduce the inter-dummy variations should be investigated in future research to improve the performed test method. Moreover, it must be evaluated if the agar dummy surface mimics the human skin to a reasonable degree. Even if the observations in both experiments aligned, more investigation is required to confirm the comparability of the two test methods.

#### 4.2.2. Electrode Construction

A general aspect to consider for knitted electrodes is the change of electrical resistance when subject to stretch [23]. This could not be observed in the performed experiments, as the electrodes were all stretched in the same amount, because they were tested on one subject only. However, when seamlessly integrating knitted electrodes into a garment, they are subject to different amounts of stretch depending on the wearer. This leads to inter-user impedance variations and must be considered in the planned applications. 

The only noticeable influence on the contact impedance variation in the performed experiments, related to the electrode construction, was found for the surface structure. The investigated uneven surface led to higher variations in contact impedance expected to be arising from a less defined contact area with the skin. Thus, from this point of view, smoother surfaces are to be preferred. Nevertheless, other types of uneven surfaces, which possess a more controlled contact area, might still be preferable, because the contact impedance is reduced by an uneven surface in dry condition. Other structures should therefore be investigated in future work.

#### 4.2.3. Pressure

A higher pressure was expected to reduce variations in effective contact area for the same electrode construction, thereby stabilizing the measured contact impedances. However, in the performed experiments, no significant differences in contact impedance variation could be found resulting from different pressure levels, and the hypothesis was rejected. Hence, applying pressure is not expected to be a suitable means to lowering contact impedance variations.

## 5. Conclusions

In conclusion, several parameters on how to reduce the contact impedance were identified. A bigger electrode and a square shape with rounded corners were found favorable to reduce the impedance. However, the effect on the stimulation selectivity and comfort for electrotherapy applications must be considered. Further, indications were found that a higher yarn density reduces the impedance as well as an uneven surface structure in case of dry electrodes. For wet electrodes, indications for an increase in impedance were observed for an uneven topography created by loops. Further, higher contact impedance variations were found for this kind of structure both in dry and wet condition, which might become problematic, especially in monitoring applications. Therefore, other uneven surface structures not consisting of loops should be investigated in future work. 

When applying pressure to the system, the same observations for the construction influence were made in the high frequency range with f > 1 kHz. Here, the electrode construction was the dominant factor for the measured contact impedance. However, below this frequency, the contact impedance was mostly determined by the applied pressure, with a higher pressure reducing the impedance, which suggests that for low frequency applications such as electrotherapy, applying pressure is a more effective means to reduce the contact impedance than changing the electrode construction. Opportunities for improving the performance of dry electrodes by applying pressure should be further investigated in future work.
